# Determinants of participation in a longitudinal two-stage study of the health consequences of the Chornobyl nuclear power plant accident

**DOI:** 10.1186/1471-2288-8-27

**Published:** 2008-05-08

**Authors:** Lin T Guey, Evelyn J Bromet, Semyon F Gluzman, Victoria Zakhozha, Vlodomyr Paniotto

**Affiliations:** 1 Spanish National Cancer Research Center (CNIO), C/Melchor Fernández Almagro 3, E-28029 Madrid, Spain; 2 Department of Psychiatry, Putnam Hall-South Campus, Stony Brook University, Stony Brook, NY, 11794-8790, USA; 3 Ukrainian Psychiatric Association, 103a Frunze Street, Kyiv, 04080, Ukraine; 4 Kiev International Institute of Sociology, vul. Voloshska 8/5, Kyiv, 04070, Ukraine

## Abstract

**Background:**

The determinants of participation in long-term follow-up studies of disasters have rarely been delineated. Even less is known from studies of events that occurred in eastern Europe. We examined the factors associated with participation in a longitudinal two-stage study conducted in Kyiv following the 1986 Chornobyl nuclear power plant accident.

**Methods:**

Six hundred child-mother dyads (300 evacuees and 300 classmate controls) were initially assessed in 1997 when the children were 11 years old, and followed up in 2005–6 when they were 19 years old. A population control group (304 mothers and 327 children) was added in 2005–6. Each assessment point involved home interviews with the children and mothers (stage 1), followed by medical examinations of the children at a clinic (stage 2). Background characteristics, health status, and Chornobyl risk perceptions were examined.

**Results:**

The participation rates in the follow-up home interviews were 87.8% for the children (88.6% for evacuees; 87.0% for classmates) and 83.7% for their mothers (86.4% for evacuees and 81.0% for classmates). Children's and mothers' participation was predicted by one another's study participation and attendance at the medical examination at time 1. Mother's participation was also predicted by initial concerns about her child's health, greater psychological distress, and Chornobyl risk perceptions. In 1997, 91.2% of the children had a medical examination (91.7% of evacuees and 90.7% of classmates); in 2005–6, 85.2% were examined (83.0% of evacuees, 87.7% of classmates, 85.0% of population controls). At both times, poor health perceptions were associated with receiving a medical examination. In 2005–6, clinic attendance was also associated with the young adults' risk perceptions, depression or generalized anxiety disorder, lower standard of living, and female gender.

**Conclusion:**

Despite our low attrition rates, we identified several determinants of selective participation consistent with previous research. Although evacuee status was not associated with participation, Chornobyl risk perceptions were strong predictors of mothers' follow-up participation and attendance at the medical examinations. Understanding selective participation offers valuable insight for future longitudinal disaster studies that integrate psychiatric and medical epidemiologic research.

## Background

Long-term follow-up studies of disaster cohorts offer insights into the enduring health and mental health effects of catastrophic events and hence provide valuable information for post-disaster public health planning. Additionally, studies that focus on children provide clues as to the long-term effects of traumatic stressors on childhood development and adjustment [1]. While most disaster studies have been cross-sectional or had follow-ups of less than 2 years [2-5], we identified 14 long-term panel studies of disaster victims with follow-up periods greater than 2 years [1,6-18]. The loss to follow-up in these studies was substantial, with the majority of these studies losing over a quarter of their original sample at later assessment points [1,6,7,10,14,16-18]. As with general population studies, failure to locate and refusal to participate each contributed to sample loss. In disaster studies, these issues are compounded if the victims are permanently evacuated from their homes and/or angry about difficulties in securing post-disaster relief. Furthermore, sudden and violent events, such as explosions and transport disasters, are likely to produce post-traumatic stress responses which may negatively influence an individual's willingness to participate [19]. Although these problems can be minimized by intensive tracking and monitoring of the sample [20], most disaster studies were initially designed as cross-sectional investigations and had no capacity to engage in sample retention activities.

Understanding the participation biases incurred by loss to follow-up is important for subsequent statistical analyses and the interpretation of results. Furthermore, insight into response patterns may offer valuable recruitment and engagement information for future studies. By and large, long-term disaster studies have reported few, if any, significant demographic or clinical predictors of follow-up participation. One important exception is the 4-year follow-up of survivors of the Netherlands fireworks disaster, which found that female gender and younger age were associated with participation and that response patterns differed for respondents of western and non-western descent [14]. Notably, they found that among non-western respondents, increased psychological symptom severity was associated with follow-up participation whereas among non-western respondents, the reverse occurred. The overall lack of significant predictors of attrition in disaster research stands in rather sharp contrast to findings from general population cohorts, which typically show that follow-up participation is associated with female gender [21-24], younger age [21,25-27], higher education [21,25,28,29], and lower levels of psychopathology [21,23,25,28,30].

For the most part, the follow-up studies noted above were conducted as single stage studies within each assessment point. However, studies of toxic exposures that have medical sequelae as well are sometimes implemented as 2-stage studies. For example, Havenaar et al. [31] evaluated the health and mental health effects of the Chornobyl disaster by administering a self-report questionnaire (stage 1) followed by a physician-administered medical and psychiatric examination with a selected high-risk and random subsample (stage 2). The response rate for stage 2 was higher for the exposed (82%) than the unexposed (65%) groups. Subjective health appraisals at stage 1 were not associated with participation at stage 2. Again, this finding stands in contrast to those of general population samples in which initial poor health perceptions and illness history significantly influence stage 2 participation [32,33].

In 1997, we conducted a 2-stage study of the psychological and medical aftermath of the Chornobyl nuclear power plant accident on children who were infants or in utero when the accident occurred in 1986 and were evacuated to Kyiv from the 30-kilometer zone around the plant. These children constitute a high risk group for thyroid cancer and have been the focus of medical, scientific, and societal monitoring since the accident occurred [34,35]. The children and their mothers were first interviewed in their homes (stage 1), and the children were then medically evaluated at a clinic (stage 2) [36]. We recently conducted an 8-year follow-up of the children and mothers using the same design. This study is one of a handful of epidemiologic studies conducted in eastern Europe to date. Given the importance of potential participation bias, we examined the demographic, maternal and self-rated health, mental health, and disaster-related attitudinal determinants of follow-up participation by the children and the mothers and the children's attendance at the medical examinations. These domains were examined because prior studies of disaster and non-disaster cohorts in western settings have illustrated their predictive value with respect to follow-up participation.

We employ the Ukrainian transliteration for Chornobyl (Chernobyl) and Kyiv (Kiev).

## Methods

### Background

On April 26, 1986, a nuclear reactor exploded at the Chornobyl nuclear power plant, located in northeastern Ukraine, releasing an unprecedented amount of radiation into the environment. The contamination was widespread, with an estimated 6.6 million people exposed to the radioactive fall-out [34]. The 30-km zone surrounding the plant was permanently evacuated, and many of the evacuees were resettled in Kyiv, which received less radioactive contamination than other areas [37].

### Sample

Six hundred children and their mothers were first studied in 1997 (time 1). The sample was comprised of two groups: 300 evacuee children and mothers and 300 classmate children and mothers. The inclusion criteria for the evacuee children were living in the 30-km zone around the power plant when the accident occurred, residing in Kyiv in 1997 when the study was conducted, and being born between February 1, 1985 and January 31, 1987. They were thus in utero to age 15 months when the accident occurred and constitute a high risk group for thyroid cancer [34,35]. The sampling frame was created by integrating the Chornobyl Registry of the Ministry of Health with lists from two large, humanitarian organizations: Help for Families from Chornobyl and Children of Chornobyl For Survival. In 1996, when the initial pilot work was conducted, there were 693 evacuee children in the targeted age range residing in Kyiv. Children were randomly selected until we reached the goal of N = 300 evacuees. Gender-matched classmates were selected as the comparison group. Up to 3 gender-matched classmates were identified from the homeroom rosters of the evacuee children in the event of refusal. The participation rates were 92% for evacuees and 85% for classmates. The median age of the evacuees and classmates was 11; 51.7% of both groups was female. The median ages of the mothers were 37 for the evacuees and 38 for the classmates.

**Figure 1:**
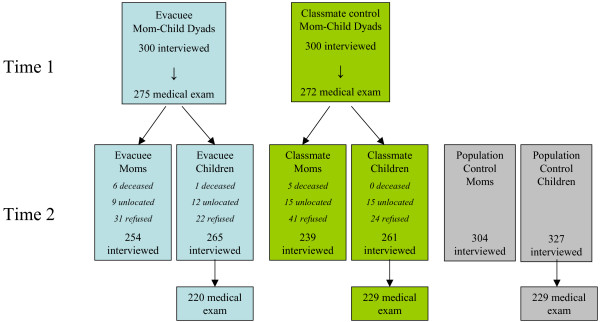
Participation at time 1 (1997) and time 2 (2005–6) by mothers and children in Kyiv.

An 8-year follow-up was conducted in 2005–6 (time 2). Eighty-eight percent of the baseline sample was living at their original address. We traced the remaining respondents by speaking with neighbors, checking with the local schools, and checking local directories in the towns where they had relocated. In the end, we located all but 27 of the children (4.5%) and 24 of the mothers (4.1%) (Figure 1).

A significant limitation of the baseline study was that the controls were restricted to the evacuee children's classmates and thus were not representative of the population of Kyiv. At time 2, we added a population control group consisting of a representative sample of young adults and their mothers in Kyiv. The eligibility criteria were (1) the young adult was born in 1985–1986, (2) the family resided in Kyiv in 1997 when the baseline study was conducted, (3) the mother was available for an interview in 1997, and (4) the family was not evacuated from a contaminated area. Sampling software was used to generate a random list of households in Kyiv, and telephone screening (97% of households in Kyiv have telephones) was used to identify eligible respondents. The response rates were 85.4% (327 out of 383 contacted) of young adults and 79.4% (304 out of 383) of mothers; there were 296 child-mother dyads.

Our follow-up analyses were therefore based on 853 young adults and 797 mothers (Figure 1). The median age of the young adult evacuees, classmates, and population controls was 19, and approximately half was female (evacuees 52.5%, classmates 51.3%, population controls 51.4%). The median ages of the mothers were 45 for the evacuees and 46 for the classmates and population controls.

### Procedure

Participants were interviewed in their homes by trained survey research personnel using a structured interview format. At baseline, the interviewer used a paper-pencil booklet; at follow-up, participants were interviewed by a computer-assisted method. After the home interviews, the children received a medical examination at a clinic in Kyiv. Considerable efforts went into engaging the sample to participate in the follow-up study and in the medical examinations, including special training of the interviewers on recruitment and conversion techniques, financial remuneration, and holding lotteries during the period of the fieldwork. At time 1, the mothers brought their children to the clinic, whereas at time 2, the young adults came to the clinic on their own. Thus, at the end of the home interview at time 2, the interviewers showed the young adult respondents a power-point presentation that included pictures of the clinic, the medical staff, a young adult having an eye examination, and the benefits of attending the clinic. The clinic staff also offered free transportation to the clinic at both assessment points.

The interviewers were employed by independent survey research firms in Kyiv (SOCIS-Gallop at time 1 and the Kiev International Institute of Sociology [KIIS] at time 2). Interviewer training lasted one week and included didactic presentations, group exercises, one-on-one practice sessions, and pilot testing. Each interviewer was observed at least once in the field, and 10% of the interviews were monitored. At time 1, the interviews lasted 45–60 minutes for the children and 2 hours for the mothers; at time 2, they lasted approximately 2.25 hours for the children and 2 hours for the mothers.

The study procedures were approved by institutional review boards of Stony Brook University and the Kiev International Institute of Sociology (KIIS). The consent forms were translated into Russian and Ukrainian. Written informed consent was obtained.

### Measures

The interviews were translated into Russian and back-translated into English following the guidelines of the World Health Organization [38]. The instruments were then translated from Russian into Ukrainian.

#### Time 1 predictors of follow-up participation and baseline clinic attendance

Four sets of predictors obtained at time 1 were examined:

##### ▪ Background characteristics

In addition to the child's gender and evacuee status, the following background characteristics were obtained from the interview with the mothers: whether the child was in utero at the time of the accident (based on date of birth) and two socio-economic indicators – perceived standard of living (Likert scale developed in Kyiv: 0 = lowest to 10 = highest) and parental education (either parent graduated from university versus less).

##### ▪ Child well-being

Three indicators of physical health were obtained from the mothers: whether the child had a medical check-up within the past year, whether the child had multiple colds (defined as ≥ 2 colds) in the past year, and overall concern about the child's health as reflect in their scores on the Children's Somatization Inventory (P-CSI, the sum of 37 items indicating symptom frequency in the past 2 weeks rated 1 = not at all to 5 = a whole lot, Cronbach's α = 0.91) [39]. One indicator of mental health was also drawn from the mothers, namely, severity of behavior problems commonly seen in pre-pubescent children (e.g., attention-deficit/hyperactivity and oppositional defiant disorder symptoms) measured with the Stony Brook Child Symptom Inventory [40]. The child's self-report on the Children's Somatization Inventory (CSI, α = 0.94) [41] and the school record of absenteeism in the first two quarters (0, 1–10 days, >10 days) were also examined.

##### ▪ Mother well-being

Both physical and emotional well-being were considered, including the mother's rating of self-assessed health (very poor/poor versus moderate/good/excellent), the Illness Worry scale (the sum of 9 items assessing hypochondriac concerns rated yes/no, α = 0.66) [42], and the Global Severity Index (GSI) of the Symptom Checklist-90 (SCL-90) (α = 0.97) [43], an overall rating of current psychological distress.

##### ▪ Mother's Chornobyl risk perceptions

Three risk perceptions were examined: diagnosed with Chornobyl-related illness (i.e., if the mother was ever told by a physician that she had an illness that was a direct consequence of Chornobyl), perception that child's health was very much affected by the accident (versus somewhat or not affected), and the Distrust of Authorities scale (mean of 7 items on distrust of governmental, media, medical, and Chornobyl-related organizations rated 1 = completely trust to 5 = completely distrust, α = 0.72) [44].

#### Time 2 predictors of clinic attendance

The following variables drawn from the interview with the young adults were examined:

##### ▪ Background characteristics

The young adult's current education (university student versus working/other) was examined, along with the background characteristics enumerated above.

##### ▪ Self-reported health

Six variables were examined: having had a medical check-up in the past year, multiple colds (defined as ≥ 2 colds) in the past year, the Illness Worry scale (α = 0.63), the CSI (α = 0.89), days out of role in the past month due to problems with physical health, mental health, or substance use (WHO Disability Assessment score dichotomized into impaired [highest quintile] versus not impaired) [45], and duration of headache-related impairment over the past year, based on a modified version of the Migraine Disability Assessment Scale (none, < 1 month, ≥ 1 month) [46].

##### ▪ Self-reported mental health

We considered the occurrence in the previous year of episodes of major depressive disorder (MDD) or generalized anxiety disorder (GAD) as diagnosed by the Composite International Diagnostic Interview (CIDI) version 3.0 [38].

##### ▪ Chornobyl risk perceptions

We examined self-reports of being diagnosed with a Chornobyl-related illness (i.e., if the respondent was ever told by a physician that he or she had an illness that was a direct consequence of Chornobyl), perceiving health as being affected by the accident (very much versus somewhat or not affected), discussing the consequences of Chornobyl with friends, family and neighbors (often versus sometimes/rarely), and the Distrust of Authorities scale (α = 0.73).

### Statistical analyses

Logistic regression analyses, adjusting for group status, were performed to examine associations between key variables and follow-up participation separately for children and mothers. The non-participant group was coded as the reference category in the logistic regression models. Interactions between group status and key variables were additionally included in the models to identify differential response patterns between evacuees and classmates. Continuous variables were standardized in logistic regression models to facilitate interpretation of the odds ratios. We also performed a stepwise backwards-elimination logistic regression analysis of the key variables and their interactions using a likelihood ratio criterion to identify a parsimonious multivariable model [47]. Similar analyses were performed to investigate the influence of key variables on attendance at the medical examination at both time points. We tested linear trends in ordinal predictors through orthogonal polynomial contrasts in logistic regression models.

## Results

### Participation in the follow-up interview

At time 2, one evacuee child died from a drowning accident, and 11 mothers (6 evacuees and 5 classmate controls) were deceased. Causes of death among the mothers were stroke (one evacuee and one classmate control), heart disease (one evacuee), cancer (3 evacuees and 3 classmate controls), multiple sclerosis (one classmate control), and unknown reasons (one evacuee). As shown in Figure 1, a total of 27 children and 24 mothers could not be located, and 46 children and 72 mothers refused participation at time 2. We were unable to stratify the analyses according to these different types of non-response due to the small sample size. However, a comparison of these non-response groups on all key variables did not reveal any significant differences (see Additional file 1). Thus, the unlocated and refused were pooled to create the non-participant group, with the deceased excluded from the analyses.

The participation rates in the follow-up study were 87.8% for the children (88.6% for evacuees; 87.0% for classmate controls) and 83.7% for their mothers (86.4% for evacuees and 81.0% for classmate controls). The participation rate for the child-mother dyads was 81.1% (evacuees: 82.9%, classmates: 79.3%).

We next examined the determinants of follow-up participation among the young adults (Table 1). Evacuee status was not significantly associated with follow-up participation. The only variables that predicted follow-up participation were having a medical examination at time 1 and mothers' participation at time 2. The interaction terms for each predictor and group status were also examined, and none was found to be statistically significant. In the final model, only maternal participation was significant. We note also that the evacuees' participation at follow-up was not significantly associated with whether the matched classmates participated (McNemar's test χ^2 ^= 0.2, df = 1, *p *> 0.05, for 300 evacuee-classmate pairs).

**Table 1:** Factors associated with children's participation at follow-up

	Participants (N = 526)^b^	Non-participants (N = 73)^b^	Adjusted for group OR (95% CI)	Multivariable model^c ^OR (95% CI)
Attended clinic at time 1	485 (92.2)	61 (83.6)	2.3 (1.2–4.7) *	--^c^
Mother participated at follow-up	477 (92.3)	16 (22.5)	41.6 (21.7–79.7) ***	46.9 (23.9–92.1) ***
				
**Background characteristics^a^**				
Evacuee status	265 (50.4)	34 (46.6)	--	--^c^
Female child	273 (51.9)	37 (50.7)	1.1 (0.6–1.7)	--^c^
Child in utero at time of accident	158 (30.0)	25 (34.2)	0.8 (0.5–1.4)	--^c^
Standard of living, mean ± SD^c^	3.8 ± 1.6	3.6 ± 1.7	1.1 (0.9–1.4)	--^c^
University graduate (either parent)	163 (31.0)	23 (31.5)	1.0 (0.6–1.8)	--^c^
				
**Child well-being^a^**				
No medical checkup in past year	343 (65.2)	44 (60.3)	1.4 (0.8–2.5)	--^c^
≥ 2 colds in past year	375 (71.6)	56 (76.7)	0.7 (0.4–1.33)	--^c^
P-CSI (mother report), mean ± SD^d^	18.6 ± 14.4	17.6 ± 13.7	1.1 (0.8–1.4)	--^c^
Childhood behavioral problems	106 (20.2)	14 (19.2)	1.1 (0.6–2.0)	--^c^
CSI (child self-report), mean ± SD^d^	16.5 ± 16.0	16.3 ± 18.7	1.0 (0.8–1.3)	--^c^
Days absent from school				
None	170 (34.1)	24 (35.3)	1.0	--^c^
1–10 days	227 (45.6)	32 (47.1)	1.0 (0.6–1.8)	--^c^
> 10 days	101 (20.3)	12 (17.6)	1.2 (0.6–2.5)	--^c^
		linear trend	χ^2^(1) = 0.2	
**Mother's Chornobyl risk perception^a^**				
Child's health perceived as very affected by Chornobyl	231 (43.9)	32 (43.8)	1.0 (0.6–1.6)	--^c^
Distrust of authorities, mean ± SD^d^	3.1 ± 0.6	3.2 ± 0.6	0.8 (0.7–1.1)	--^c^

^a ^All variables are based on maternal reports except the child-rated CSI and the school record information on absences. No interactions between these variables and evacuee status were found to be statistically significant.

^b ^Values are numbers (percentage, calculated from total column) except when noted.

^c ^The final multivariable model was obtained following a stepwise backwards likelihood-ratio elimination procedure.

^d ^Continuous variables were standardized in logistic regression models so that the ORs may be interpreted as the odds of participation for a one standard deviation increase in the predictor.

* *p *< 0.05; ** *p *< 0.01; *** *p *< 0.001

Table 2 shows the relationship of the same variables with the mothers' participation in the follow-up. As noted, evacuee status was not significantly associated with mothers' participation. In addition, as with the children, mothers who brought their children to the medical clinic at time 1 were more likely to participate, and there was a strong association between child and mother participation at time 2. Mothers were also twice as likely to participate if their children had not had a medical check-up in the year preceding time 1. Additionally, mothers who expressed more concern about their own health on the Illness Worry scale and more distress on the GSI were more likely to participate at follow-up. Lastly, women who reported at time 1 that a doctor diagnosed them as having an illness caused by Chornoby were nearly twice as likely to participate in the follow-up. None of the interaction terms between each of the predictors and group was statistically significant. Five of these six statistically significant predictors remained in the final multivariable model using the step-wise backwards-elimination procedure. In descending order of magnitude based on the adjusted odds ratios, these variables were children's participation at follow-up, children's participation in the medical examination, mother diagnosis of a Chornobyl-related illness, children's not having a medical check-up, and higher GSI scores.

**Table 2:** Factors associated with mothers' participation at follow-up

	Participants (N = 493)^b^	Non-participants (N = 96)^b^	Adjusted for group OR (95% CI)	Multivariable model^c ^OR (95% CI)
Child attended clinic in 1997	464 (94.1)	73 (76.0)	5.0 (2.8–9.2) ***	6.7 (3.1–14.4) ***
Child participated at follow-up	477 (96.8)	40 (42.1)	41.6 (21.7–79.7) ***	61.3 (28.8–130.5) ***
				
**Background characteristics^a^**				
Evacuee status	254 (51.5)	40 (41.7)	--	--^c^
Female child	254 (51.5)	51 (53.1)	0.9 (0.6–1.5)	--^c^
Child in utero at time of accident	149 (30.2)	29 (30.2)	1.0 (0.6–1.6)	--^c^
Standard of living, mean ± SD^d^	3.8 ± 1.6	3.9 ± 1.7	1.0 (0.8–1.2)	--^c^
University graduate (either parent)	149 (30.3)	34 (35.4)	0.9 (0.5–1.4)	--^c^
				
**Child well-being^a^**				
No medical checkup in past year	326 (66.1)	54 (56.3)	2.1 (1.3–3.5) **	2.1 (1.1–3.9) *
≥ 2 colds in past year	363 (73.9)	65 (67.7)	1.3 (0.8–2.1)	--^c^
P-CSI (mother report), mean ± SD^d^	19.1 ± 14.3	16.2 ± 13.9	1.2 (0.9–1.5)	--^c^
Childhood behavioral problems	103 (20.9)	15 (15.6)	1.4 (0.8–2.6)	--^c^
CSI (child self-report), mean ± SD^d^	16.6 ± 15.5	16.9 ± 20.3	1.0 (0.8–1.2)	--^c^
Days absent from school				
None	156 (33.3)	35 (39.8)	1.00	--^c^
1–10 days	214 (45.7)	40 (45.5)	1.2 (0.7–2.0)	--^c^
> 10 days	98 (20.9)	13 (14.8)	1.6 (0.8–3.3)	--^c^
		linear trend	χ^2^(1) = 2.0	
**Mother well-being^a^**				
Rate health as poor	155 (31.5)	25 (26.0)	1.2 (0.7–2.0)	--^c^
Illness worry, mean ± SD^d^	3.3 ± 2.2	2.8 ± 2.0	1.3 (1.0–1.6) *	--^c^
SCL-90 GSI, mean ± SD^d^	0.8 ± 0.5	0.6 ± 0.4	1.4 (1.1–1.8) **	1.6 (1.1–2.3) *
				
**Mother's Chornobyl risk perception^a^**				
Diagnosed with Chornobyl-related illness	192 (38.9)	23 (24.0)	1.9 (1.1–3.2)*	2.9 (1.4–5.9) **
Child's health perceived as very affected by Chornobyl	224 (45.4)	35 (36.5)	1.3 (0.8–2.1)	--^c^
Distrust of authorities, mean ± SD^d^	3.1 ± 0.6	3.2 ± 0.5	0.9 (0.7–1.1)	--^c^

^a ^All variables are based on maternal reports except the child-rated CSI and the school record information on absences. No interactions between these variables and evacuee status were found to be statistically significant.

^b ^Values are numbers (percentage, calculated from total column) except when noted.

^c ^The final multivariable model was obtained following a stepwise backwards likelihood-ratio elimination procedure.

^d ^Continuous variables were standardized in logistic regression models so that the ORs may be interpreted as the odds of participation for a one standard deviation increase in the predictor.

* *p *< 0.05; ** *p *< 0.01; *** *p *< 0.001

### Participation in the medical examinations in 1997

As shown in Figure 1, 547 children (91.2%) were examined at the clinic (275 evacuee children [91.7%] and 272 classmate control children [90.7%]) in 1997. The majority of these children (69.0%) had their medical examinations within one month following the interview. Evacuee status was clearly not associated with clinic attendance. Table 3 shows that not having a medical check-up in the year preceding time 1 and having multiple colds in that time period increased the likelihood of attendance two-fold. The examined children also had higher self-reported CSI scores than those who were not examined. Mothers who reported greater distrust in authorities were less likely to bring their child to the clinic. While mothers' P-CSI was not significantly associated with participation adjusting for group, there was a significant interaction of P-CSI with group indicating that in the evacuee sample, mothers' P-CSI scores were not associated with bringing their child to the clinic, but in the classmate sample, higher P-CSI scores were significantly associated with clinic attendance. In the multivariable model, P-CSI and the interaction term for P-CSI and group were statistically significant, as were all other predictors described above apart from multiple colds. We note that attendance of the matched classmate pair was not associated with evacuee attendance (McNemar χ^2 ^= 0.7, df = 1, *p *> 0.05, for 300 evacuee-classmate pairs).

**Table 3:** Factors associated with children's participation in the medical examination at time 1

	Examined (N = 547)^b^	Not examined (N = 53)^b^	Adjusted for group OR (95% CI)	Multivariable model^c ^OR (95% CI)
**Background characteristics^a^**				
Evacuee status	275 (50.3)	25 (47.2)	--	--^c^
Female child	287 (52.5)	23 (43.4)	1.4 (0.8–2.5)	--^c^
Child in utero at time of accident	166 (30.3)	18 (34.0)	0.8 (0.5–1.5)	--^c^
Standard of living, mean ± SD^d^	3.8 ± 1.6	3.8 ± 2.0	1.0 (0.7–1.3)	--^c^
University graduate (either parent)	166 (30.4)	20 (37.7)	0.7 (0.4–1.3)	--^c^
				
**Child well-being^a^**				
No medical checkup in past year	361 (66.0)	27 (50.9)	2.4 (1.2–4.5) **	2.6 (1.4–4.9) **
≥ 2 colds in past year	400 (73.4)	32 (60.4)	1.8 (1.0–3.2) *	--^c^
P-CSI (mother report), mean ± SD^d^	18.5 ± 14.1	18.4 ± 16.2	1.8 (1.0–3.2)^e^	2.0 (1.1–3.7) *
Childhood behavioral problems	108 (19.7)	12 (22.6)	0.8 (0.4–1.7)	--^c^
CSI (child self-report), mean ± SD^d^	17.0 ± 16.6	11.4 ± 11.0	1.7 (1.1–2.8) *	1.7 (1.1–2.7) *
Days absent from school				
None	179 (34.5)	16 (33.3)	1.0	--^c^
1–10 days	237 (45.7)	22 (45.8)	1.0 (0.5–1.9)	--^c^
> 10 days	103 (19.8)	10 (20.8)	0.9 (0.4–2.1)	--^c^
		linear trend	χ^2^(1) = 0.05	
**Mother well-being^a^**				
Rate health as poor	172 (31.4)	13 (25.0)	1.4 (0.7–2.6)	--^c^
Illness worry, mean ± SD^d^	3.3 ± 2.2	2.9 ± 2.1	1.2 (0.9–1.6)	--^c^
SCL-90 GSI, mean ± SD^d^	0.8 ± 0.5	0.7 ± 0.4	1.3 (0.9–1.8)	--^c^
				
**Mother's Chornobyl risk perception^a^**				
Child's health perceived as very affected by Chornobyl	239 (43.7)	25 (47.2)	0.8 (0.5–1.5)	--^c^
Distrust of authorities, mean ± SD^d^	3.1 ± 0.5	3.3 ± 0.6	0.7 (0.5–0.9) **	0.6 (0.5–0.9) **
				
**Statistically significant interactions with group^a^**				
P-CSI (mother report) X group (evacuee status)^d^			0.4 (0.2–0.9) *	0.4 (0.2–0.8) **

^a ^All variables are based on maternal reports except the child-rated CSI and the school record information on absences.

^b ^Values are numbers (percentage, calculated from total column) except when noted.

^c ^The final multivariable model was obtained following a stepwise backwards likelihood-ratio elimination procedure.

^d ^Continuous variables were standardized in logistic regression models so that the ORs may be interpreted as the odds of participation for a one standard deviation increase in the predictor.

^e^Adjusted for group and the interaction term; *p *= 0.07

* *p *< 0.05; ** *p *< 0.01; *** *p *< 0.001

### Participation in the medical examinations in 2005–6

A total of 727 young adults (85.2% of the 853 participants) attended the clinic, including 220 evacuees (83.0%), 229 classmate controls (87.7%), and 278 population controls (85.0%). Similar to time 1, the majority of the young adults (65.1%) had their medical evaluations within one month after their interview, and group status was not a significant predictor. Those whose mothers participated in the interview were more than six times as likely to receive a medical examination. Females and those with a lower standard of living were also significantly more likely to attend. The young adults' subjective health assessments and Chornobyl risk perceptions significantly influenced their decision to attend the clinic. Specifically, those who perceived their health as poor during the home interview, had higher somatization scores on the CSI, were more worried about their health, reported greater impairment, and were depressed or anxious were significantly more likely to have a medical evaluation. Moreover, being told by a doctor that they had an illness caused by Chornobyl, believing that their health was adversely affected by Chornobyl, engaging in frequent discussions about Chornobyl, and having lower distrust in authorities increased the likelihood of having a medical evaluation. No significant interactions between key predictors and group were found. Three variables were statistically significant in the final multivariable model: mothers' participation at follow-up, frequent discussion about Chornobyl, and lower distrust in authorities. Gender, standard of living, and depression/generalized anxiety disorder were retained in the model but with *p *values 0.05 – 0.10.

In the panel sample, those who were examined in 1997 were almost three times as likely to be examined again in 2005–6 (Table 4). Finally, attendance of the matched classmate pair was not associated with evacuee attendance (McNemar χ^2 ^= 2.3, df = 1, *p *> 0.05, for 230 evacuee-classmate pairs).

**Table 4:** Factors associated with young adult's participation in the medical examination at time 2

	Examined (N = 727)^b^	Not examined (N = 126)^b^	Adjusted for group OR (95% CI)	Multivariable model^c ^OR (95% CI)
Had medical examination at time 1^d^	420 (93.5)	65 (84.4)	2.8 (1.3–5.7) **	--^d^
Mother participated at follow-up	683 (93.9)	90 (71.4)	6.4 (3.9–10.5) ***	6.1 (3.7–10.2) ***
				
**Background characteristics at follow-up^a^**				
Group status				
Evacuee	220 (30.3)	45 (35.7)	--	--^c^
Classmate control	229 (31.5)	32 (25.4)	--	--^c^
Population control	278 (38.2)	49 (38.9)	--	--^c^
Female	389 (53.5)	52 (41.3)	1.9 (1.1–3.1) *	1.4 (0.9–2.2)
In utero at time of accident	235 (32.3)	46 (36.5)	0.8 (0.6–1.3)	--^c^
Standard of living, mean ± SD^e^	5.6 ± 1.4	5.9 ± 1.4	0.8 (0.7–1.0) *	0.8 (0.7–1.0)
Either parent graduated from university	277 (40.6)	30 (33.3)	1.3 (0.8–2.2)	--^c^
Young adult attends university	473 (65.1)	78 (61.9)	1.2 (0.8–1.7)	--^c^
				
**Self-reported physical health at time 2^a^**				
No medical checkup in past year	491 (67.8)	91 (72.2)	0.8 (0.5–1.2)	--^c^
≥ 2 colds in past year	429 (59.0)	72 (57.1)	1.1 (0.7–1.6)	--^c^
Illness worry, mean ± SD^e^	2.3 ± 1.9	1.9 ± 1.8	1.3 (1.0–1.6) *	--^c^
CSI, mean ± SD^e^	14.1 ± 11.0	10.8 ± 10.1	1.5 (1.2–1.8) **	--^c^
Days out of role, health-related impairment	153 (21.0)	16 (12.7)	1.9 (1.1–3.3) *	--^c^
Headache-related impairment				
None	417 (57.4)	91 (72.2)	1.0	--^c^
< 1 month	235 (32.3)	30 (23.8)	1.7 (1.1–2.7) *	--^c^
≥ 1 month	75 (10.3)	5 (4.0)	3.4 (1.4–8.8) *	--^c^
		linear trend	χ^2 ^(1) = 6.7 **	
**Self-reported mental health at time 2^a^**				
MDD/GAD (CIDI diagnosis)	118 (16.2)	9 (7.1)	2.5 (1.2–5.1) *	2.0 (1.0–4.2)
				
**Young adults' risk perceptions at time 2^a^**				
Diagnosed with a Chornobyl-related illness	166 (22.8)	18 (14.3)	1.9 (1.1–3.2) *	--^c^
Health perceived as very affected by Chornobyl	110 (15.1)	10 (7.9)	2.2 (1.1–4.4) *	--^c^
Discuss consequences of disaster often	92 (12.7)	4 (3.2)	4.6 (1.7–12.9) **	4.1 (1.4–11.7) **
Distrust of authorities, mean ± SD^e^	3.0 ± 0.6	3.1 ± 0.6	0.8 (0.6–0.9) **	0.8 (0.6–0.9) *

^a ^All variables are derived from the young adult's self-reports with the exception of parental education. No interactions between these variables and group status were found to be statistically significant.

^b ^Values are numbers (percentage, calculated from total column) except when noted.

^c ^The final multivariable model was obtained following a stepwise backwards likelihood-ratio elimination procedure.

^d ^This predictor is only applicable to the original cohort assessed in 1997 (N = 265 evacuees; N = 261 classmate controls) and thus was excluded from the multivariable model in order to retain the data from the population control group.

^e ^Continuous variables were standardized in logistic regression models so that the ORs may be interpreted as the odds of participation for a one standard deviation increase in the predictor.

* *p *< 0.05; ** *p *< 0.01; *** *p *< 0.001

## Discussion

This study examined the determinants of participation in a multi-stage long-term follow-up cohort study of children and mothers exposed to the Chornobyl nuclear power plant disaster. Despite our relatively low attrition rate (12.2% in the children and 16.3% in the mothers), we identified several significant determinants of loss to follow-up. The variables predicting follow-up participation by the children were having a medical evaluation at time 1 and mothers' participation in the follow-up study, e.g., greater maternal investment in the study. The participation by the mothers was influenced not just by whether their children participated, but also by their initial health concerns about their child, their own illness worries and psychological distress, and their Chornobyl risk perceptions. Surprisingly, evacuee status was not a significant predictor of participation in the follow-up study. It is possible that the even though the classmate controls were not immediately exposed to the disaster, they still considered themselves to be affected by the Chornobyl accident and thus were motivated to participate in the follow-up study.

The results also showed that participation in the medical examinations at each time point was influenced by poor health perceptions and trust in authority figures, but not by evacuee status as Havenaar et al. had observed [31]. It is conceivable that because our sample consisted of mothers and their young children that the majority of the mothers, irrespective of group status, encouraged their children to take advantage of the free medical examination at both assessment points. Also, although evacuee status did not predict clinic attendance, we did observe a differential clinic attendance pattern between evacuees and classmates at time 1. Specifically, higher somatization scores, as rated by the mothers, were associated with clinic attendance at time 1 for classmates only. It is probable that this pattern was not observed in evacuee mothers because their children were exposed to potentially harmful toxic agents, and thus they did not need this extra incentive to bring their children to the clinic. The classmate mothers, on the other hand, were less concerned about Chornobyl-related health consequences and were more inclined to bring their child to the clinic if they believed that their child had higher levels of somatic symptoms.

At follow-up, when the young adults brought themselves to the clinic, having a medical examination at time 1, reporting a lower standard of living, greater health problems, illness-related functional impairment, depression or anxiety, and Chornobyl risk perceptions were significant determinants of clinic attendance. In the analysis that adjusted for group status, we also found that females were more likely to attend the clinic, consistent with health care utilization studies that have shown that females are more likely to use medical services than males [48,49]. Some have attributed this difference to an increased sensitivity to symptoms and overall interest in health by females [48,49]. The backwards elimination analysis showed that the young adults' clinic attendance was primarily influenced by mothers' participation and by the young adults' trust in authorities and frequency of discussing Chornobyl with family and peers. It is interesting that at time 1, the mothers' distrust in authority was negatively associated with bringing their children to the clinic, and at follow-up, the young adults' own distrust in authority was negatively associated with attendance.

It is important to ground our findings in the context of the limitations of our study. Because of the relatively small sample size, we were unable to conduct stratified attrition analyses according to the differing types of non-response (deceased, unlocated, and refused). However, we found no significant differences between those unlocated and refused on all variables examined in this report. Also, the study was conducted in a single city (Kyiv, Ukraine) in the aftermath of a specific event. Because all disasters have unique characteristics, the findings do not necessarily generalize to other events, such as natural disasters and explosions in urban areas. However, as described below, our findings were indeed reasonably consistent with findings from prior research. Lastly, because of the extensive efforts to encourage participation that were implemented throughout the fieldwork, our attrition rates were relatively low and thus our statistical power to detect significant determinants of participation was reduced.

The present study sheds light on three points. First, we found that higher levels of psychological distress and health concerns were associated with participation by the mothers in Kyiv. This finding is consistent with the results on attrition in the non-western immigrants participating in Dutch fireworks disaster cohort [14,50]. It is also consistent with findings from a longitudinal general population study in North America by Psaty et al. [51], who found that poor health status was a significant determinant of participation among non-western immigrant respondents. As noted earlier, attrition findings from western population health studies generally find the reverse, that is, increased psychopathology is associated with non-participation [21,23,25,28,30]. It has been hypothesized that non-western immigrants might believe that participation is only meaningful in the presence of health problems [14]. An alternative explanation is that following traumatic events involving toxic exposures that have the potential to cause cancer and other fatal diseases, increased symptomatology, particularly anxiety symptoms stemming from somatic preoccupation, would increase participation in clinical research. Thus while Weisaeth et al. [19] observed in their study of individuals exposed to an industrial explosion that non-participation can be associated with post-traumatic stress disorder due to the presence of avoidance symptoms, after a toxic exposure like Chornobyl, hypervigilance may in fact be one of the dominant psychological mechanisms that leads people to participate in clinical research.

Second, consistent with previous studies [32,33], illness history and poor health perceptions had a strong influence on attendance in the medical examination stage of the study at both time points. In fact, early in the course of the fieldwork, we became concerned about the low rate of clinic attendance at time 2. After eliciting the reasons why the young adults were not coming to the clinic, we developed strategies for improving recruitment. For example, in addition to the power point presentation at the end of the interview, the interviewers were instructed to emphasize the free eye examination and that if a serious health problem were detected, the clinic would provide or locate the best available treatment in Kyiv. Despite all of our best efforts, the results showed that there was still some selective non-participation in the medical examination stage of the follow-up study although the recruitment rate improved over time and conceivably the bias was attenuated.

Third, perhaps the most compelling finding of this study is the pervasive influence of disaster risk perceptions on participation. Mothers who reported that a doctor diagnosed them with an illness caused by Chornobyl were more likely to participate in the follow-up study. Furthermore, young adults who reported that a doctor diagnosed them with an illness caused by Chornobyl, believed their health to be substantially affected by the disaster and discussed the consequences of the disaster often were more likely to have a clinical evaluation at follow-up. It is interesting to note that in the focus group discussions conducted prior to the follow-up study, the young adults had expressed boredom with the topic of Chornobyl and stated that the issue was solely a concern of their mothers. However, as illustrated by our empirical findings, these young adults (either subconsciously or consciously) were motivated to have a medical examination because of their Chornoby risk perceptions.

A comprehensive investigation of attrition is critical for the analysis and interpretation of any follow-up cohort study. We intend to apply the findings from the current set of analyses in our future longitudinal evaluations of the health outcomes of the Chornobyl accident using statistical missing data techniques such as weighting and multiple imputation.

## Conclusion

Long-term studies of disaster cohorts are needed to investigate the enduring health and mental health consequences of a disaster and for planning long-term post-disaster health and mental health interventions. Relatively few studies have examined the factors associated with the long-term psychological influence of disasters, and even fewer have focused on children [10,15-17]. Moreover, there is a dearth of such studies conducted in eastern European settings. In designing such studies, obtaining high participation rates can only be achieved by anticipating and understanding the causes of selective participation. This study identified significant determinants of participation that were consistent with previous research. Our results have implications for longitudinal studies of toxic disasters that integrate psychiatric and medical epidemiologic research and for researchers who wish to conduct studies in an eastern European environment.

## Competing interests

The authors declare that they have no competing interests.

## Authors' contributions

LTG performed all the statistical analyses and drafted the manuscript. EJB revised the manuscript and was the principal investigator of the project. SG supervised the medical examination stage of the study. VZ supervised all aspects of the field work, including translation/back translation, training and monitoring of interviewers, and selection of population controls. VP designed sampling method for population controls and as the director of KIIS was responsible for the overall conduct of the study. All authors read and approved the final manuscript.

## Pre-publication history

The pre-publication history for this paper can be accessed here:



## Supplementary Material

Additional file 1 Descriptive characteristics of the non-participation groups. Summary statistics of all key variables for the non-participation groups. Click here for file
