# Supplementary material for: Determinants of participation in a longitudinal two-stage study of the health consequences of the Chornobyl nuclear power plant accident
Source: BMC Med Res Methodol. 2008 May 8;8:27. doi: 10.1186/1471-2288-8-27 (PMC2396662; doi:10.1186/1471-2288-8-27)
Supplement: Additional file 1 — Descriptive characteristics of the non-participation groups. Summary statistics of all key variables for the non-participation groups. [file 1471-2288-8-27-S1.doc]

**Additional file 1**: Descriptive characteristics of the non-participation groups

|  | **CHILDREN** | |  | **MOTHERS** | | |  |
| --- | --- | --- | --- | --- | --- | --- | --- |
|  | Unlocated N=27 | Refused N=46 | χ^2^ | Deceased N=11 | Unlocated N=24 | Refused N=72 | χ^2 a^ |
|  | N (%) | N (%) |  | N (%) | N (%) | N (%) |  |
|  |  |  |  |  |  |  |  |
| **Background characteristics** |  |  |  |  |  |  |  |
| Evacuee status | 12 (44.4) | 22 (47.8) | 0.1 | 6 (54.5) | 9 (37.5) | 31 (43.1) | 0.2 |
| Female child | 11 (40.7) | 26 (56.5) | 1.7 | 5 (45.5) | 13 (54.2) | 38 (52.8) | 0.01 |
| Child in utero at time of accident | 9 (33.3) | 16 (34.8) | 0.01 | 6 (54.5) | 5 (20.8) | 24 (33.3) | 1.3 |
| Standard of living, mean±SD | 3.3±1.6 | 3.8±1.8 | *t*=-1.4 | 2.8±1.9 | 3.4±1.6 | 4.0±1.7 | t=-1.4 |
| University graduate (either parent) | 11 (40.7) | 12 (26.1) | 1.7 | 3 (27.3) | 11 (45.8) | 23 (31.9) | 1.5 |
|  |  |  |  |  |  |  |  |
| **Child’s well-being** |  |  |  |  |  |  |  |
| No medical checkup in past year | 14 (51.9) | 15 (32.6) | 2.6 | 3 (27.3) | 11 (45.8) | 31 (43.1) | 0.06 |
| ≥ 2 colds in past year | 22 (81.5) | 34 (73.9) | 0.5 | 4 (36.4) | 20 (83.3) | 45 (62.5) | 3.6 |
| P-CSI (mother report), mean±SD | 16.3±12.6 | 18.4±14.4 | *t*=-0.6 | 12.4±12.8 | 15.4±12.3 | 16.5±14.5 | *t*=0.8 |
| Childhood behavioral problems | 6 (22.2) | 8 (17.4) | 0.3 | 2 (18.2) | 5 (20.8) | 10 (13.9) | 0.7 |
| CSI (child self-report), mean±SD | 19.2±24.1 | 14.6±14.6 | *t*=1.0 | 10.5±8.5 | 21.2±25.3 | 15.4±18.3 | *t*=1.2 |
| Days absent from school |  |  |  |  |  |  |  |
| None | 8 (33.3) | 16 (36.4) | 3.6 | 4 (36.4) | 9 (42.9) | 26 (38.8) | 2.5 |
| 1-10 days | 9 (37.5) | 23 (52.3) |  | 5 (45.5) | 7 (33.3) | 33 (49.3) |  |
| > 10 days | 7 (29.2) | 5 (11.4) |  | 2 (18.2) | 5 (23.8) | 8 (11.9) |  |
|  |  |  |  |  |  |  |  |
| **Mother’s well being** |  |  |  |  |  |  |  |
| Rate health as poor | 8 (30.8) | 17 (37.0) | 0.3 | 5 (45.5) | 6 (25.0) | 19 (26.4) | 0.2 |
| Illness worry, mean±SD | 3.0±2.2 | 3.0±2.1 | *t*=0.03 | 3.0±2.4 | 3.3±2.1 | 2.7±1.9 | *t*=1.3 |
| SCL-90 GSI, mean±SD | 0.7±0.5 | 0.8±0.4 | *t*=-0.4 | 0.8±0.6 | 0.8±0.6 | 0.6±0.4 | *t*=1.3 |
|  |  |  |  |  |  |  |  |
| **Mother’s Chornobyl risk perception** |  |  |  |  |  |  |  |
| Diagnosed with Chornobyl-related illness | 8 (29.6) | 17 (37.0) | 0.4 | 4 (36.4) | 7 (29.2) | 16 (22.2) | 0.5 |
| Child’s health perceived as affected by Chornobyl | 13 (48.1) | 19 (41.3) | 0.3 | 5 (45.5) | 8 (33.3) | 27 (37.5) | 0.1 |
| Distrust of authorities scale, mean±SD | 3.3±0.6 | 3.1±0.5 | *t*=1.1 | 2.8±0.5 | 3.2±0.7 | 3.2±0.5 | *t*=0.4 |

^a^ Test statistics compare the unlocated and refused. The deceased were excluded from all subsequent analyses.
